# Propane Dehydrogenation Catalyzed by Pt Clusters (Pt_2_–Pt_6_) in Gas Phase and Supported on g‑C_3_N_4_ and γ‑Al_2_O_3_: A Theoretical Study

**DOI:** 10.1021/acsomega.5c07626

**Published:** 2025-09-25

**Authors:** Jie Pan, Gerard Bru, Jorge J. Carbó, Cyril Godard, Josep M. Ricart

**Affiliations:** Departament de Química Física i Inorgànica, Universitat Rovira i Virgili, C/ Marcel·lí Domingo 1, 43007 Tarragona, Spain

## Abstract

Density functional theory (DFT) investigations and microkinetic analyses were conducted to explore the propane dehydrogenation (PDH) process catalyzed by small platinum clusters (Pt_2_–Pt_6_). γ-Al_2_O_3_ and graphitic carbon nitride (g-C_3_N_4_) were considered as support materials to evaluate their influence on the catalytic performance. Among the studied clusters, Pt_5_ demonstrated superior PDH activity, selectivity, and stability. Notably, the presence of supports significantly enhanced the selectivity of the reaction, with the Pt_5_/g-C_3_N_4_ system exhibiting the most favorable performance.

## Graphical abstract

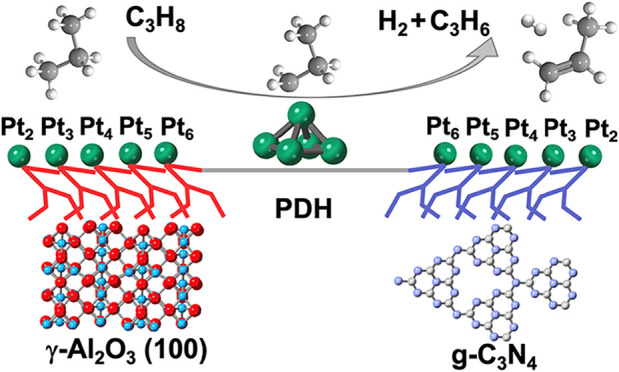

## Introduction

The production of light olefins, including ethylene, propylene, and butene, is highly important to the global chemical industry and plays a critical role in the production of a vast array of everyday products. Driven by sustained economic growth, a rising global population, and expanding industrial output, the demand for these critical feedstocks continues to increase. Among them, propylene stands out due to its exceptional versatility and wide range of applications. It serves as a key precursor for the manufacture of numerous high-value chemicals and materials, such as polypropylene, acetone, acrylonitrile, acrolein, acrylic acid, acrylates, and propylene oxide. As a result of this surging demand, an increasingly pronounced supply-demand gap has emerged, emphasizing the need for efficient and sustainable production routes for propylene.^1,2^

In response, various methodologies have been developed for propylene production, such as the methanol-to-olefins, the Fischer–Tropsch-to-olefins process, and the propane dehydrogenation (PDH).^3–9^ Among these, PDH is especially promising owing to its straightforward feed–product relationship and economical use of propane.^1,10^ Furthermore, hydrogen, generated as a valuable byproduct, increases the interest in this process. Industrial implementation typically requires high temperatures (550–750 °C) and low partial pressures (∼0.1 MPa) due to the highly endothermic nature of the reaction (Δ*H* ^0^ = 123.8 kJ mol^–1^ at 298.15 K), so robust and highly active catalysts are needed to efficiently cleave the C–H bonds of propane.^11^

Conventionally, platinum- and chromium-based catalysts have been employed for PDH.^1,12,13^ Chromium oxides, while cost-effective, pose environmental and health hazards and suffer rapid deactivation via side reactions.^1^ In contrast, platinum-based systems offer superior environmental compatibility and greater resistance to deactivation, although they are still susceptible to side reactions such as deep dehydrogenation and coke formation.^12–14^ Enhancing their performance requires fine-tuning both activity and selectivity, which can be addressed by controlling catalyst morphology and support interactions.^14–16^

Cluster size is a critical factor that influences catalytic behavior. Experimentally, Zhu and Chen et al.^17^ have shown that small Pt clusters dominated by low-coordination metal atoms exhibit enhanced C–H bond activation, while larger clusters with Pt(111) facets favor weaker binding of propene, improving selectivity. Computed catalytic activities of various Pt*_n_* clusters (*n* = 2–6) reveal different C–H activation energies (*E*_a_) for the first bond breaking of propane, indicating the effect of cluster size on catalytic activity.^18^ However, this study lacked an analysis of key factors such as the second C–H bond cleavage and product desorption energies. Therefore, it is pertinent to investigate these aspects.

Another essential strategy involves tailoring the interaction between the metal clusters and suitable supports. Metal–support interactions not only influence cluster dispersion and stability but also can modulate electronic properties to improve catalytic performance. In an experimental study, Vajda and Curtiss et al.^19^ deposited size preselected Pt_8–10_ clusters on porous aluminum oxide and reported a 40-fold increase in activity compared with the conventional catalyst for the oxidative dehydrogenation of propane, while maintaining high selectivity to propene. They also use DFT to study PDH on a Pt_4_ cluster and attribute the high activity to the low coordination of Pt within the clusters, favoring the C–H bond breaking over that of C–C or C=C bonds.^19^

Supported clusters have consequently attracted significant attention as effective catalysts for PDH. In particular, γ-Al_2_O_3_ is considered one of the most versatile oxide supports due to its remarkable porosity.^20–22^ Moreover, Al^3+^ penta-coordinated sites (Al_p_) on the (100) facets of γ-Al_2_O_3_ are favorable anchoring points for Pt clusters, mitigating sintering and improving durability.^23^ Therefore, the γ-Al_2_O_3_(100) surface is selected as a reference for anchoring Pt*_n_*, compared with the naked Pt*_n_* subnanometric clusters, and further investigates the impact of oxide supports on Pt*_n_* clusters’ properties toward PDH.

Additionally, supports like graphene, doped graphene, or graphitic carbon nitride (g-C_3_N_4_) have emerged as promising nonoxide alternatives. Thus, it is pertinent to compare with nonoxide supports such as graphene. However, pristine graphene is not suitable as a support under high-temperature reaction conditions due to the tendency of metal clusters to diffuse and aggregate on its surface.^24^ Consequently, research efforts have turned toward modifying graphene through the introduction of vacancies and heteroatoms, a strategy that effectively incorporates g-C_3_N_4_, which combines both structural features. The morphology of g-C_3_N_4_ resembles fragments of graphene interconnected by tertiary amines, accompanied by uniformly distributed holes and abundant lone pairs of electrons originating from nitrogen atoms.^24–27^

Within our previous computational studies on PDH by Pt catalysts, reactive ReaxFF simulations found that in the stepped Pt(211) facet, propane activation occurs in low coordinated Pt atoms at the step, resulting in a more reactive and selective surface topology than Pt(111) and Pt(100) ones.^28^ Thus, we expect that small clusters with low coordinated morphologies would lead to highly active and selective catalysts. Indeed, we performed DFT calculations and ab initio molecular dynamics (AIMD) simulations on the Pt_1_ atom and the Pt_4_ clusters supported on g-C_3_N_4_. However, both systems showed some limitations in PDH catalysis. In the single-atom (Pt_1_/g-C_3_N_4_) system, the Pt atom can diffuse below the surface of the support, while in the single-cluster (Pt_4_/g-C_3_N_4_) one, deep dehydrogenated species cover the active sites, lowering the activity.^29^

In this study, we advance the understanding of factors that optimize propane dehydrogenation in platinum-based catalysts by examining the influence of cluster size and support material. Specifically, we perform a theoretical study of small platinum clusters (Pt_2_ to Pt_6_), both naked and supported on γ-Al_2_O_3_ and g-C_3_N_4_, evaluating how these variables influence catalytic performance.

## Computational Details

All calculations were performed using the Vienna Ab Initio Simulation Package (VASP 5.3.5), with the same settings as in our previous work.^29,30^ Projector augmented wave (PAW) pseudopotentials were used to describe the core electron density and its interaction with the valence electron density.^31^ The exchange–correlation interactions were calculated using the PBE-generalized gradient approximation (GGA) functional.^32^ The van der Waals interactions were considered by the DFT-D3 method with zero-damping of Grimme.^33^ Spin polarization and dipole correction were always included. The residual force and energy convergence criteria were set to 1 × 10^–3^ eV/Å and 1 × 10^–5^ eV, respectively. A plain wave energy cutoff of 400 eV for the valence electron expansion was selected, and a Monkhorst–Pack mesh with a 2 × 2 × 1 k-point grid was used to sample the Brillouin zone, in accordance with previous papers on Pt*_n_*/γ-Al_2_O_3_(100) and further tested for g-C_3_N_4_.^23,29^ A 2 × 2 × 1 supercell (14.246 × 14.250 × 22 Å^3^ for g-C_3_N_4_ and 16.826 × 11.174 × 28 Å^3^ for γ-Al_2_O_3_) was used to avoid unwanted interactions between repeated slabs. A cube of 15 × 15 × 15 Å^3^ with a k-point grid of 1 × 1 × 1 was used for isolated Pt atoms and naked clusters (Pt*_n_*). The transition states (TS) of elementary reactions were obtained by the dimer method with the input files referring to a previous study.^29^ The proper character of the local minima and the TS has been confirmed by vibrational frequencies analysis, ensuring a single imaginary frequency for every TS, and the absence of imaginary frequencies for reactants, intermediates, and products.

In addition, Bader’s atoms-in-molecules electronic density analysis^34^ was also carried out to obtain atomic charges via integration of electronic density within regions delimited by zero flux surfaces, allowing for the evaluation of the charge transfer between species.

The cohesive energies (*E*_coh_) of Pt particles were calculated as follows:

1 $$E_{\mathrm{coh}}=\left(E_{{\mathrm{Pt}}_{n}}-nE_{\mathrm{Pt}}\right)/n$$

where *n* is the number of Pt atoms, *E*_Pt*_n_*_ stands for the energy of Pt*_n_*; *E*_Pt_ is the energy of gas phase Pt in its ground state.

The interaction energy (*E*_int_) of Pt with the surfaces was calculated by

2 $$E_{\mathrm{int}}=\left(E_{\mathrm{Pt}@\mathrm{support}}-E_{\mathrm{support}}-nE_{\mathrm{Pt}}\right)/n$$

where *E*_Pt@support_ is the energy of the anchored metal on the support. *E*_support_ is the energy of the support.

The adsorption energy for each adsorbed species is calculated by eq 3.

3 $$E_{\mathrm{ads}}=E_{\mathrm{mol}‐\mathrm{Pt}@\mathrm{support}}-\left(E_{\mathrm{mol}}+E_{\mathrm{Pt}@\mathrm{support}}\right)$$

where *E*_mol‑Pt@support_ is the energy of the whole system; *E*_mol_ stands for the energy of the adsorbed molecule in the gas phase; and *E*_Pt@support_ is the energy of the catalyst. The energy profiles were made with reference to propane in the gas phase and the clean surface. Besides the thermodynamic treatment, the microkinetic modeling (MKM) was also used to study the kinetic properties of the catalysts, and both details are in the Supporting Information.

## Results and Discussion

### Structure of Pt*_n_* Clusters (*n* = 2–6)

First, we optimized the bare clusters, taking into account both 2D and 3D configurations (Figure 1). Note that the 2D configurations appear to be the most stable, as described by Da Silva et al.^35^ As shown in Figure 1, the Pt–Pt bond distances range from 2.330 Å to 2.613 Å, consistent with previous theoretical and experimental findings.^36,37^ For clusters with the same number of Pt atoms, the cohesive energies (*E*_coh_, Figure S1) are similar, but 2D configurations are slightly more stable than 3D ones, with the difference being larger for Pt_6_.^35,38^ Our results are also in agreement with previous reports,^35^ which described that planar configurations were more stable for small Pt clusters, while 3D configurations became more favorable for clusters larger than Pt_10_. The *E*_coh_ increases with the cluster size and gradually converges to the bulk value (−511 kJ/mol), see Figure S1.^39^

**Figure 1:**
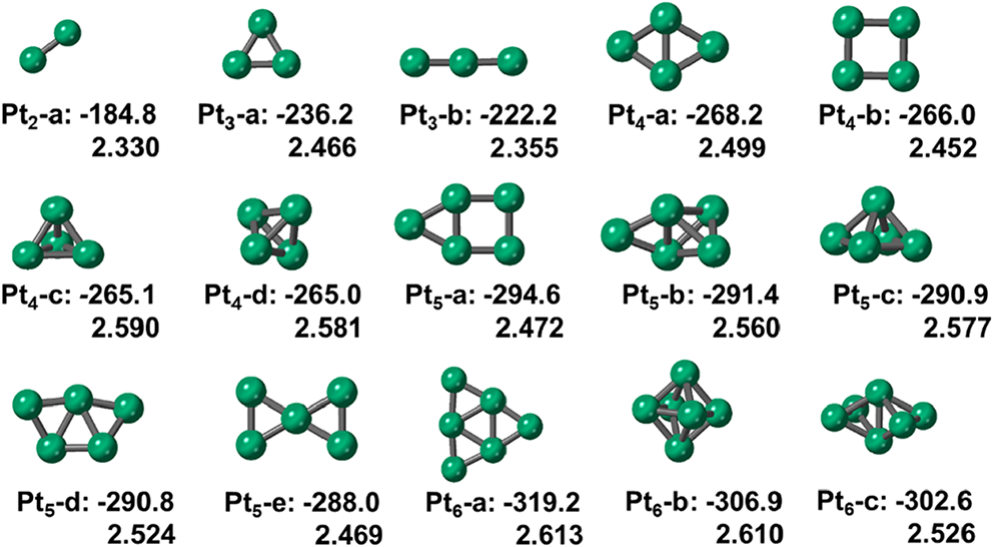
Side views of the bare Pt*_n_* clusters (*n* = 2–6). The cohesive energies (*E*_coh_) in kJ/mol (top) and average Pt–Pt bond distances in Å (bottom) are indicated.

### Pt*_n_* (*n* = 2–6) Clusters Supported on γ-Al_2_O_3_

Next, γ-Al_2_O_3_-supported Pt*_n_* clusters were examined. Previous studies demonstrated that Al^3+^ sites (Al_p_) residing on (100) facets exhibit the highest affinity for capturing Pt atoms, so the γ-Al_2_O_3_(100) surface was selected (Figure 2a).^23,40^

**Figure 2:**
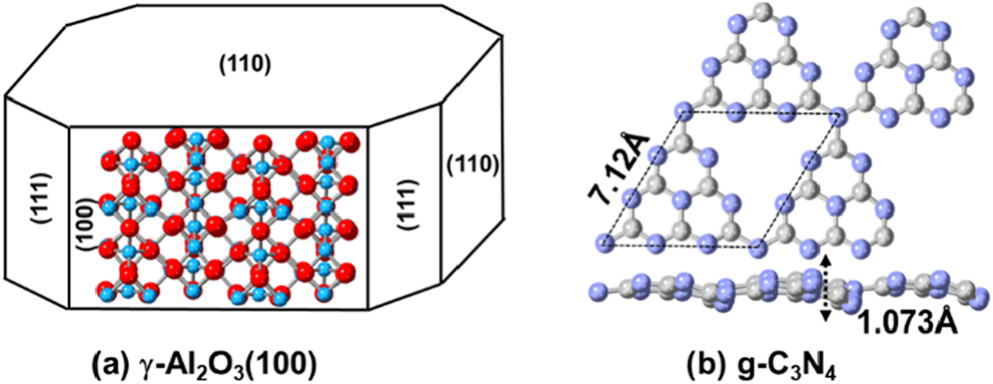
(a) Top view of γ-Al_2_O_3_(100) and (b) top and side views of g-C_3_N_4_. Al, O, C, and N are in blue, red, gray, and lavender, respectively.

The dehydrated γ-Al_2_O_3_(100) facet was used as described in the model proposed by Digne and Sautet et al.^41,42^ When the adsorption of the Pt*_n_* clusters on γ-Al_2_O_3_(100) (Figure 3) was examined, distortion on the surface was observed due to the electronic exchange between the support and clusters, which was previously documented.^43,44^ The interaction energies (*E*_int_) initially decreased with increasing cluster size, but the differences gradually diminished, and for Pt_5_ and Pt_6_, the difference became negligible. Additionally, for some configurations, the most favorable ones were not those corresponding to the adsorption of planar clusters, such as the structure of AL-**Pt_4_-d**. This can be attributed to the fact that the morphology of the supported clusters is controlled by the competition of the metal–surface and metal–metal interactions.^36^ By further comparison of the *E*_coh_ (which evaluates the aggregation capacity of the clusters, Figure 1) and *E*_int_ (which evaluates the binding energy between the cluster and the support, Figure 3), we observe that the *E*_int_ values are consistently more negative than the *E*_coh_, meaning that the clusters interact strongly with alumina and are less prone to sinter. Furthermore, the difference between *E*_int_ and *E*_coh_ slowly decreases with increasing cluster size.

**Figure 3:**
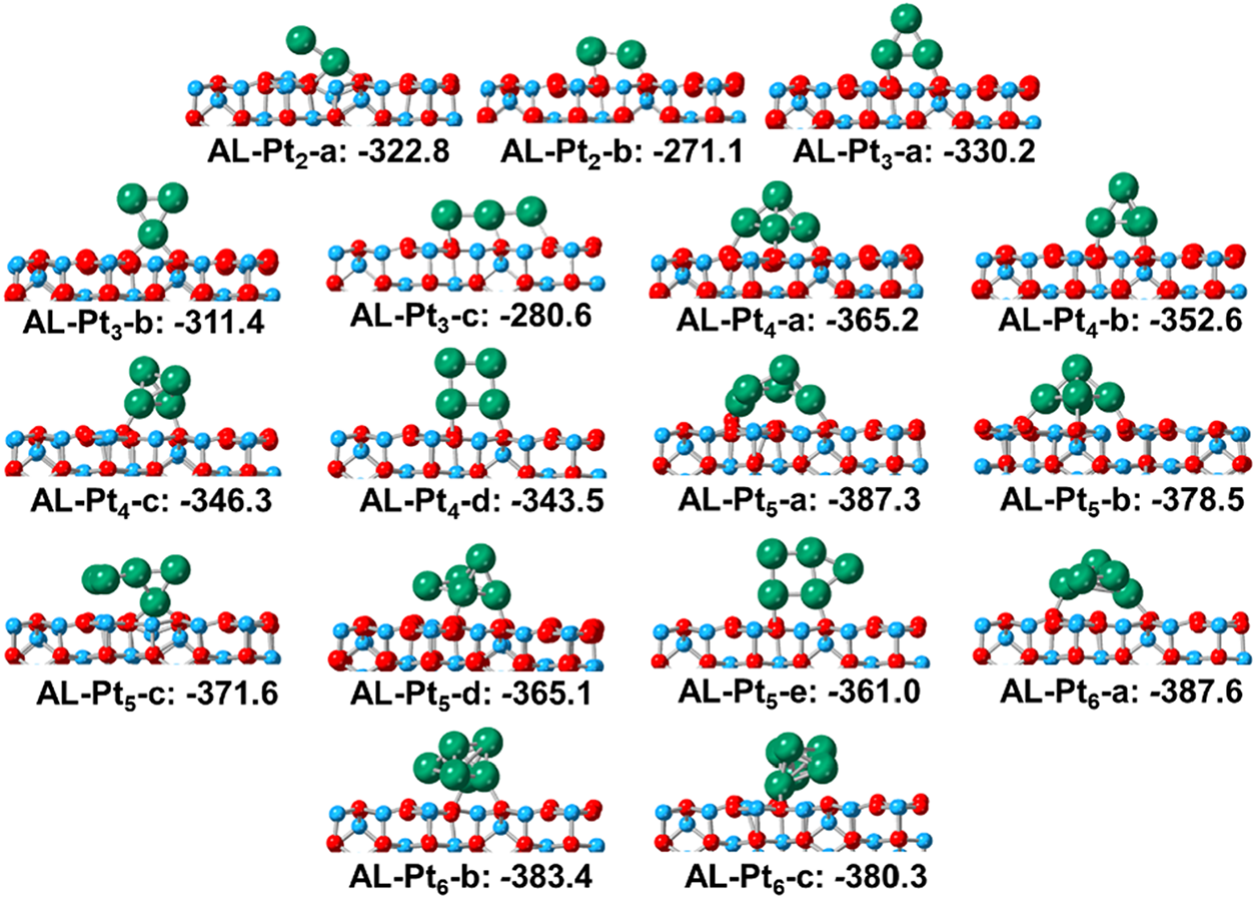
Side views of Pt*_n_*/γ-Al_2_O_3_(100). Al, O, and Pt atoms are in blue, red, and green, respectively. The interaction energies (*E*_int_, kJ/mol) between clusters and the surface are indicated. AL stands for the alumina support.

### Pt*_n_* (*n* = 2–6) Clusters Supported on g-C_3_N_4_

Next, we analyze the structures and interaction energies of Pt clusters supported on g-C_3_N_4_, whose structure and lattice were taken from our previous study,^29^ see Figure 2b. The configurations of all of the Pt*_n_* clusters adsorbed on g-C_3_N_4_ are given in Figure 4. The results were similar to those of Pt*_n_*/γ-Al_2_O_3_(100). The most stable adsorbed configuration is not always planar, as observed in the case of Pt_5_ clusters, where the **Pt**_**5**_ **-c** structure shows the largest interaction energy. The cluster–support interaction becomes stronger as cluster size increases until it reaches the maximum strength for Pt_5_. The *E*_int_ is always greater (in absolute value) than the cluster *E*_coh_.

**Figure 4:**
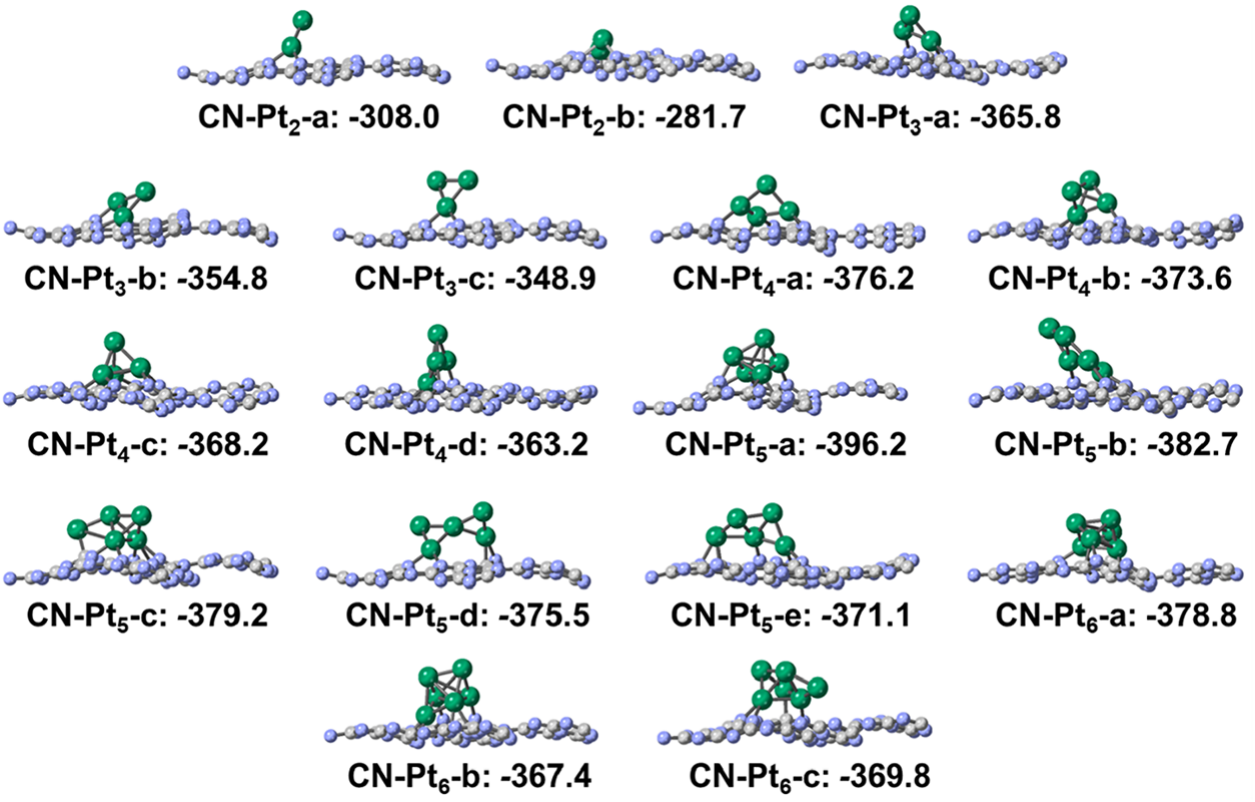
Side views of Pt*_n_*/g-C_3_N_4_. C, N, and Pt atoms are in gray, lavender, and green, respectively. The interaction energies (*E*_int_, kJ/mol) are indicated. CN stands for carbon nitride support.

Comparing the results for the two supports (Figures 3 and 4), we observe qualitatively similar interaction energies, which, depending on cluster size, are larger for the γ-Al_2_O_3_(100) or the g-C_3_N_4_ supports. For each cluster composition (Pt_2–6_), the structure with the largest calculated interaction energy is labeled as **Pt** *_**n**_* **-a**, but we did not identify any obvious tendency or the same type of cluster for both types of support. Therefore, for the sake of simplicity (also due to their similar *E*_int_ and *E*_coh_ along the series of studied clusters compared to the most stable ones), we selected the following regular Pt*_n_* clusters: linear ones for Pt_2_ (**Pt**_**2**_ **-a**, **AL-Pt**_**2**_ **-a**, **CN-Pt**_**2**_ **-a**), triangular ones for Pt_3_ cluster (**Pt**_**3**_ **-a**, **AL-Pt**_**3**_ **-a**, **CN-Pt**_**3**_ **-a**), pyramid ones for Pt_4_ (**Pt**_**4**_ **-c**, **AL-Pt**_**4**_ **-b**, **CN-Pt**_**4**_ **-c**), square pyramid ones for Pt_5_ (**Pt**_**5**_ **-c**, **AL-Pt**_**5**_ **-b**, **CN-Pt**_**5**_ **-a**), and octahedral ones for Pt_6_ (**Pt**_**6**_ **-b**, **AL-Pt**_**6**_ **-b**, **CN-Pt**_**6**_ **-b**) were selected to study the catalytic performance for PDH.

### Catalytic Performance of Bare and Supported Pt*_n_* Clusters (*n* = 2–6) in PDH

The stability of catalysts is one of the main factors to evaluate the performance of heterogeneous catalysts, which is expressed as the balance between *E*_coh_ and *E*_int_.^39^ Although it has been briefly discussed above, a further comparison is made in Figure 5a. We can see that *E*_coh_ is always weaker than *E*_int_ on both supports, indicating that adsorption is more favorable than cluster aggregation for all the clusters and that the *E*_coh_ decreases as the cluster size increases, converging toward the bulk value.^39^ For Pt*_n_*/γ-Al_2_O_3_(100), the surface–cluster interaction strengthens as cluster size increases. Moreover, the downward trend gradually weakens, resulting in only a 4.9 kJ/mol difference between Pt_5_ and Pt_6_; for Pt*_n_*/g-C_3_N_4_, similar results are detected for Pt_2_ to Pt_5_, but for Pt_6_, the *E*_int_ is stronger by 28.8 kJ/mol compared with Pt_5_, since only a maximum of 4 Pt atoms can interact with N atoms due to the size of the g-C_3_N_4_ hole. Finally, for Pt_2_ to Pt_5_, the adsorption energy on g-C_3_N_4_ is stronger than on γ-Al_2_O_3_(100), while for Pt_6_, it is the opposite, due to the change in *E*_int_ of Pt_6_ on g-C_3_N_4_.

**Figure 5:**
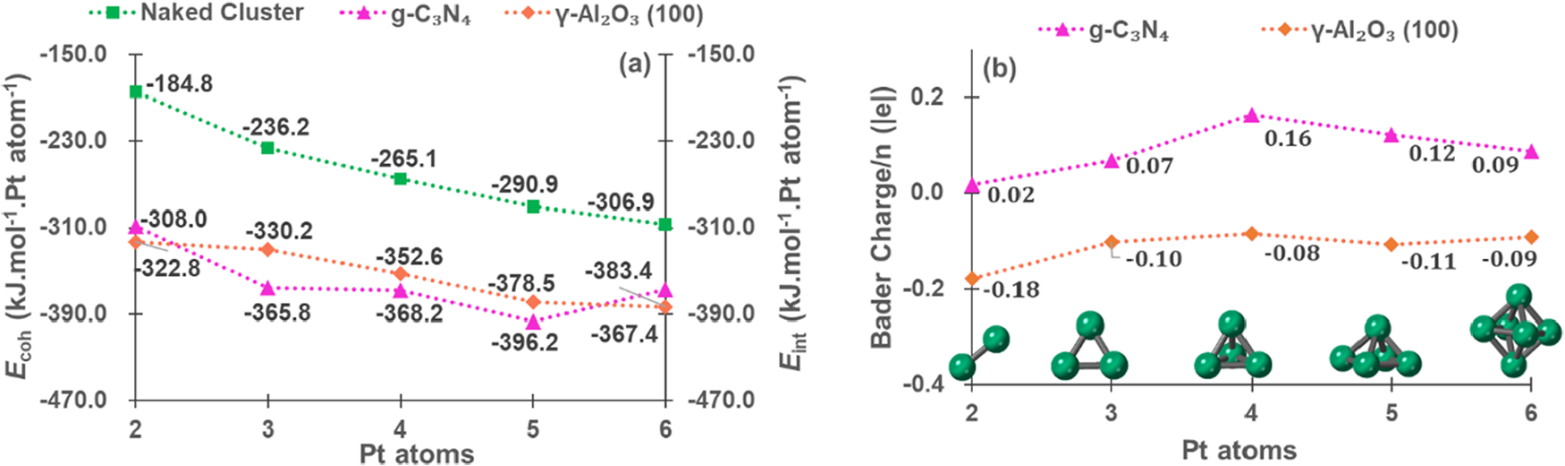
(a) *E*_coh_ of bare Pt*_n_* clusters and *E*_int_ between Pt*_n_* and γ-Al_2_O_3_(100) or g-C_3_N_4_, all energies in kJ/mol. (b) Bader charge of naked and supported Pt*_n_* clusters (mean charge per Pt atom) as a function of cluster size, the charge for each Pt atom is shown in Table S1.

The atomic charge was analyzed using the Bader approach, as shown in Figure 5b. As a reference, the Bader charges of bulk α-PtO_2_ were calculated. The computed charge on the Pt atoms was 1.4 |e|, close to the value recently reported by Wang et al. of 1.6 |e|.^45^ The atomic charges of Pt atoms in the bare neutral clusters are, as expected, close to zero. The charges of γ-Al_2_O_3_(100)-supported clusters are slightly negative, in agreement with the results reported by Sautet et al.^39^ The charges of the supported clusters on g-C_3_N_4_ are slightly positive, similar to the case of metal species anchored on N-doped carbon materials.^46,47^ However, as the size of clusters increases, the charge transfer between clusters and both supports decreases, setting atomic charges close to zero.

As the initial step of PDH, we computed the adsorption of propane on all of the catalysts. Adsorption through either primary carbon (C_1_) or secondary (C_2_) led to similar results (Figure 6a,b, respectively): (1) Propane, as expected, exhibits a weak interaction with *E*_ads_ ranging from −75.1 to −12.4 kJ/mol for all catalysts; (2) the naked clusters are more active than the supported ones, especially Pt_3_ and Pt_4_; and (3) the *E*_ads_ on clusters supported on γ-Al_2_O_3_(100) and g-C_3_N_4_ are similar.

**Figure 6:**
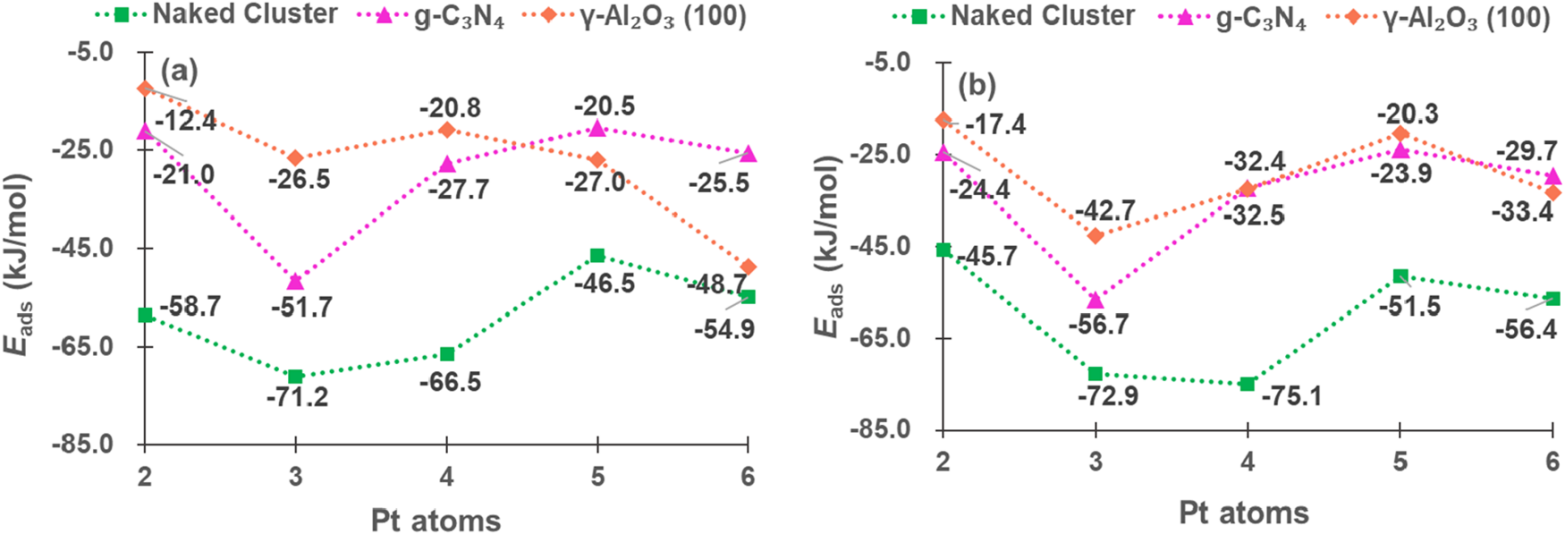
*E*_ads_ (kJ/mol) of propane, C_1_ adsorption (a) and C_2_ adsorption (b) on isolated clusters and supported on g-C_3_N_4_ and γ-Al_2_O_3_(100).

We also evaluated the adsorption energies of propene (Figure 7a) and hydrogen (Figure 7b), which are related to the selectivity of PDH. As displayed in Figure 7a, the bare clusters adsorb propene more strongly, particularly Pt_3_, Pt_4_, and Pt_6_, while for Pt_2_ and Pt_5_, the *E*_ads_ are lower; the adsorption energies on the supported clusters generally weaken as cluster size increases, reaching the weakest point for Pt_5_, while they strengthen significantly for Pt_6_. Additionally, the *E*_ads_ of propene is similar on both types of supports, with those on Pt_6_ being the stronger ones.

**Figure 7:**
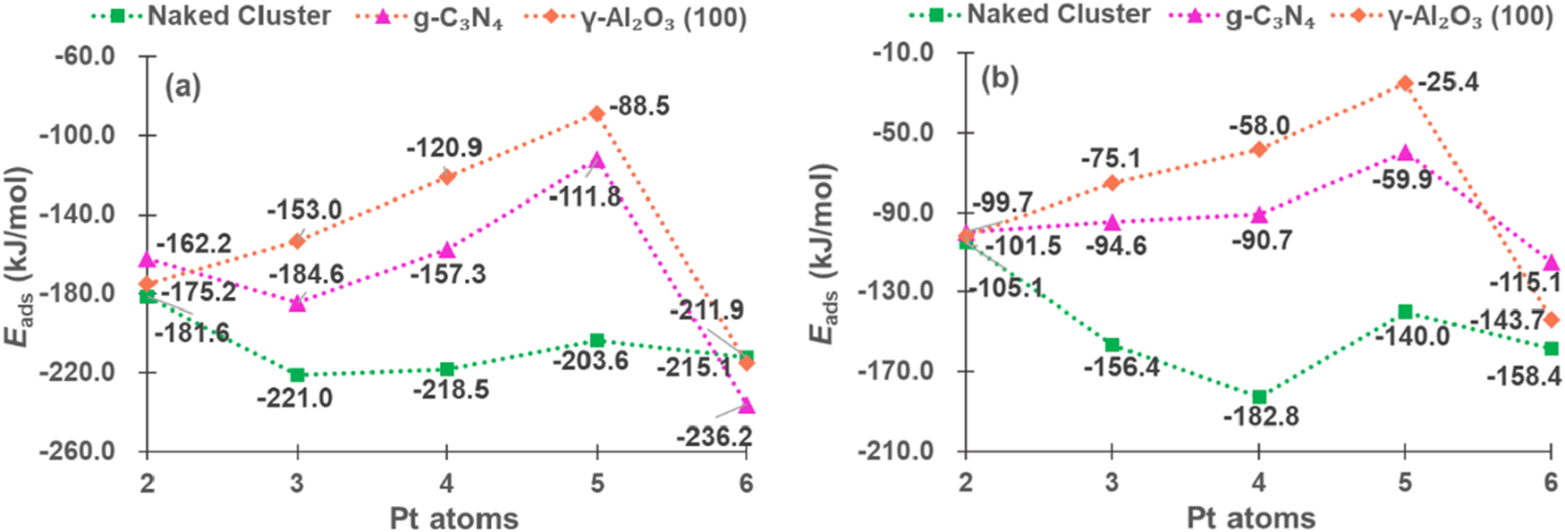
*E*_ads_ (kJ/mol) of propene (a) and hydrogen (b).

Figure 7b shows that for hydrogen adsorption on naked clusters, the same trend as propene adsorption is observed, *E*_ads_ is higher on Pt_3_, Pt_4_ and Pt_6_, being lower on Pt_2_ and Pt_5_; on γ-Al_2_O_3_(100) and g-C_3_N_4_, the *E*_ads_ weakens with cluster size from Pt_2_ to Pt_5_ but strengthens for Pt_6_, and Pt_5_ shows relatively low adsorption energy for all the systems.

Previous studies have demonstrated that both geometric and electronic factors affect the catalytic performance of Pt nanoparticles, with electronic factors becoming more dominant when the particles are smaller than 10 nm.^48,49^ Thus, the projected densities of states (pDOS in Figure 8) for Pt_5_/g-C_3_N_4_ and Pt_6_/g-C_3_N_4_ were analyzed to investigate the strong adsorption behavior of supported Pt_6_ toward propene and hydrogen. As shown in Figure 8, the supported Pt_6_ cluster exhibits more unpaired electrons (highlighted by circles) compared to Pt_5_, which facilitates molecular adsorption. In Pt_5_ (Figure 8b), only one unpaired electron is located on the top Pt atom, leading to weaker adsorption of propene and hydrogen. In contrast, Pt_6_ (Figure 8d) features two unpaired electrons on its top Pt atom, which enhances π interactions with propene and promotes chemisorption of hydrogen, thereby resulting in higher adsorption energies. In addition, the supported Pt_6_ NPs are expected to exhibit significantly stronger adsorption capability due to an additional exposed Pt atom, consistent with previous findings that the catalytic activity of Pt particles increases with the number of Pt atoms located at faces and corners.^49^ Thus, Pt_6_ was not further considered in subsequent analyses because of the high adsorption energies of propene and hydrogen, which can reduce the PDH performance.

**Figure 8:**
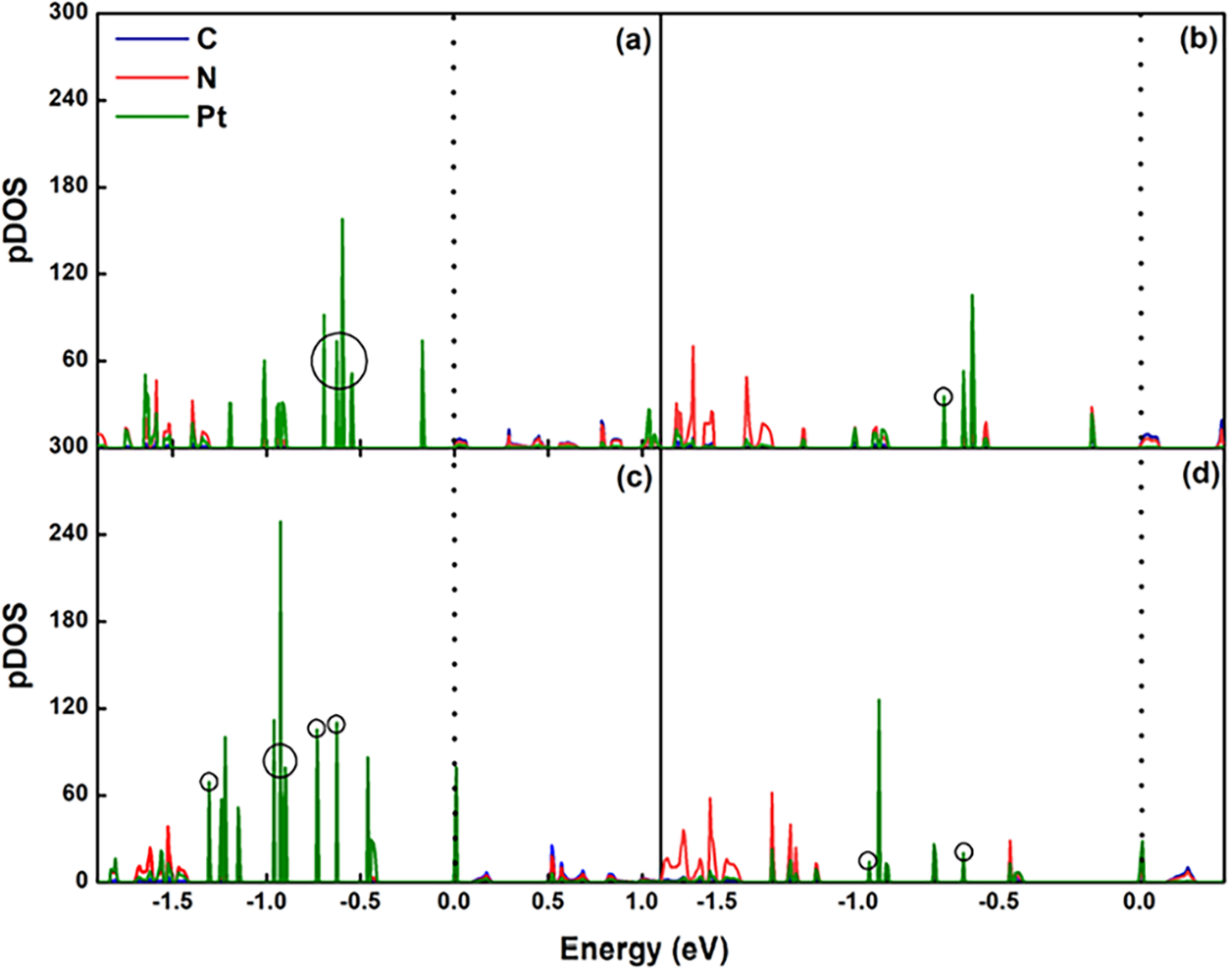
pDOS for Pt_5_/g-C_3_N_4_ (a, b) and Pt_6_/g-C_3_N_4_ (c, d), where (a) and (c) included all C, N, and Pt atoms, and (b) and (d) included all C and N, only the top Pt atom interacting with the molecule. Energy is referred to Fermi energy, and the band gap of g-C_3_N_4_ is 1.6 eV from our previous study.^29^

The performance of clusters (from Pt_2_ to Pt_5_) was further investigated by the comparison of *E*_a_ (Figure 9 for C_1_ adsorption and Figure S2 for C_2_ adsorption) in the breakage of two C–H bonds of propane. For the first C–H bond breaking, all catalysts show low energy barriers, especially for bare Pt_3_ and Pt_4_, which are nearly barrierless. In this step, Pt_5_ revealed slightly less activity than the others. These findings are similar to the results described by Ge et al.^18^ for the C–H activation of propane using a molecular model of Pt*_n_* (*n* = 2–6) (Figure S3). For the second C–H bond, the scission on all isolated clusters is almost barrier-free (below 5.0 kJ/mol). In the case of g-C_3_N_4_, the activation energies (Figure 9) first increased with size and then decreased, with the highest barrier for Pt_3_/g-C_3_N_4_. However, on γ-Al_2_O_3_(100), Pt_3_ provided the lowest barrier, which increased gradually with the cluster size.

**Figure 9:**
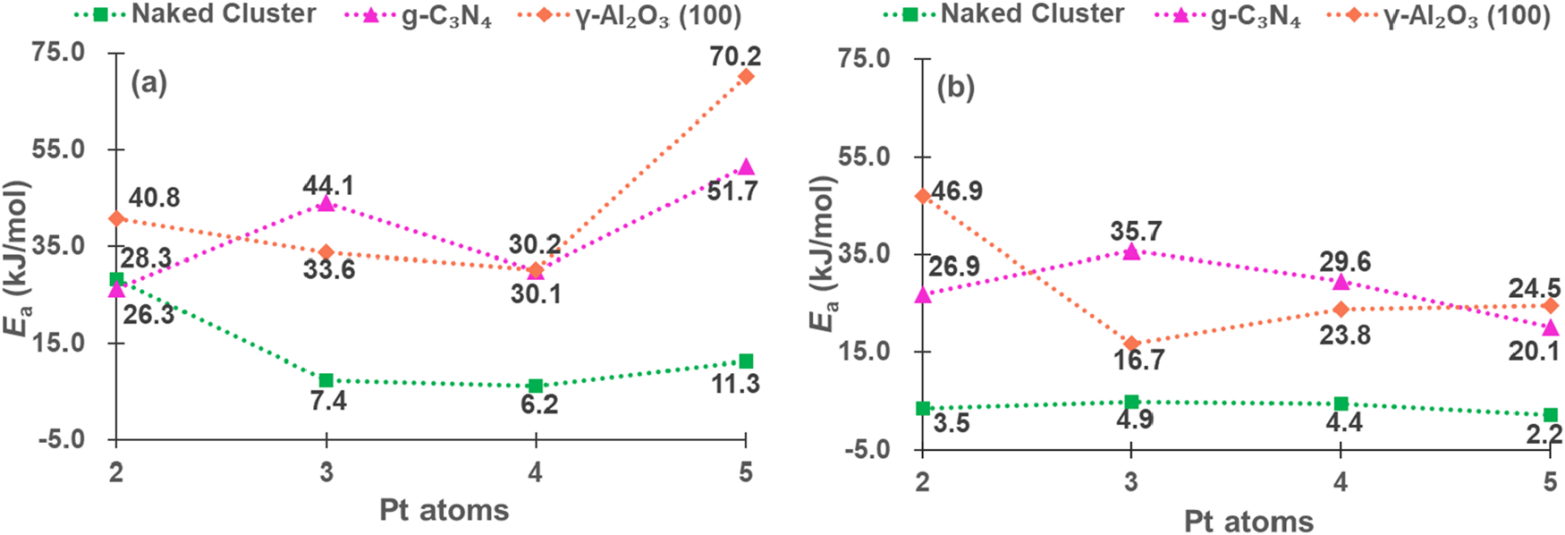
*E*_a_ (kJ/mol) for the first (a) and second (b) C–H bond cleavage of propane (C_1_ adsorption).

Based on the information above, as all clusters show high activities toward C–H cleavage, the factors determining the catalytic performance should be the stability and selectivity, with Pt_5_ presenting slightly better values.

### Free Energy Profiles for PDH on Bare and Supported Pt_5_

Herein, the free energy profiles were computed at 873.15 K (C_1_ and C_2_ adsorption are shown in Figures 10 and S4, respectively). Since the results of C_1_ and C_2_ are similar, only C_1_ adsorption is discussed. As displayed in Figure 10, the calculated adsorption energy of propane was 123.8 kJ/mol, which can be attributed to the entropic factor. Then, the first C–H bond of propane is activated with a low energy barrier of 17.2 kJ/mol, forming the first intermediate (C_3_H_7_* + H*), after which the adsorbed hydrogen atom migrates toward another Pt atom, and the hydrogen atom tends to migrate to another Pt atom, leading to a more stable intermediate (1st H migration). Subsequently, the second C–H bond is activated by the same Pt atom with an extremely low barrier of 3.4 kJ/mol, leading to π-adsorbed propene (C_3_H_6_* + 2H*). Then, the adsorbed hydrogen atom moves to another Pt atom (2nd H migration); the *E*_ads_ values for different configurations are shown in Table S2. H_2_ and propene desorb with 50.7 and 16 kJ/mol, respectively. Thus, the rate-determining step (RDS) of this process should be the desorption, which requires a total energy of 66.7 kJ/mol at 873.15 K. This can be attributed to the strong interactions between the highly active clusters and the adsorbates, highlighting the importance of supports for catalyst modification.

**Figure 10:**
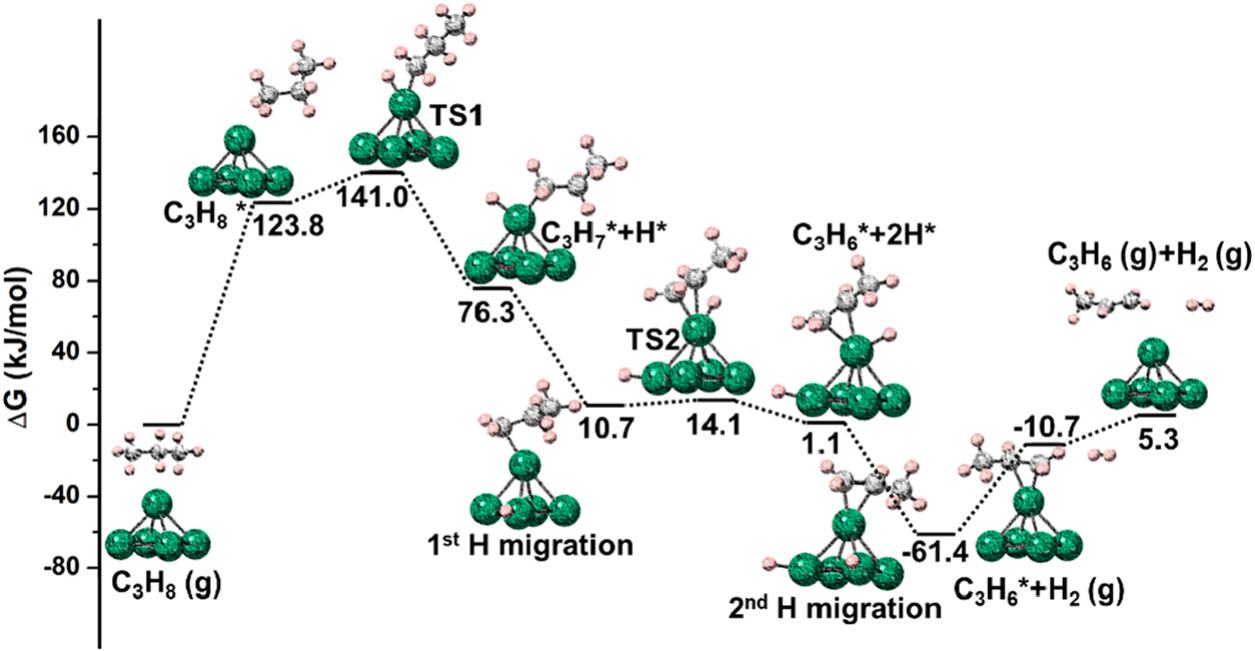
PDH free energy profile on Pt_5_ at 873.15 K, for C_1_ adsorption. C, H, and Pt atoms are in gray, pink, and green, respectively.

Then, the energy profiles of Pt_5_/γ-Al_2_O_3_(100) under the same conditions as Pt_5_ are given in Figure 11 (C_1_) and Figure S5 (C_2_). The adsorption energy of propane (157.5 kJ/mol) is 33.7 kJ/mol less favorable than for the naked Pt_5_ cluster, due to the reduced activity of supported clusters (Figures 6 and 9). Thus, the *E*_a_ (37.1 kJ/mol) for the first C–H bond activation is higher (by 17.2 kJ/mol) than for the naked Pt_5_ cluster, showing a lower activity for the C–H cleavage. The intermediate after hydrogen migration (1st H migration) is slightly less stable (by 3.9 kJ/mol) than C_3_H_7_* + H*. However, we assume that the migration occurs to regenerate the top Pt atom and activate the second C–H bond, which is consistent with a previous study on Pt_4_-catalyzed PDH.^50^ Again, the *E*_a_ (24.3 kJ/mol) for the second bond breaking is higher (by 20.9 kJ/mol) than that of the naked cluster, showing a reduced activity. On the positive side, due to the reduced adsorption energies on Pt_5_/γ-Al_2_O_3_(100), the desorption of hydrogen and propene is exergonic at 873.15 K, releasing 11.6 and 92.9 kJ/mol, respectively, and indicating an enhanced selectivity on the γ-Al_2_O_3_(100) support. Throughout the energy profile, the C–H bond cleavage (TS1) is the RDS for Pt_5_/γ-Al_2_O_3_(100).

**Figure 11:**
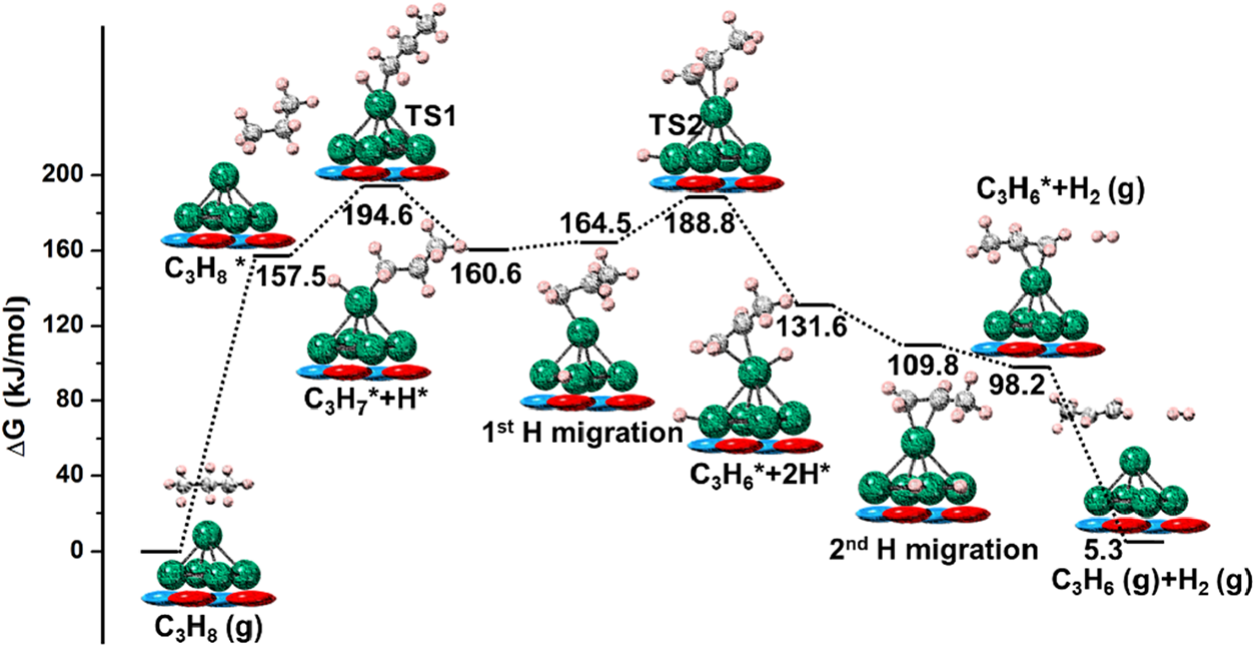
PDH free energy profile on Pt_5_/γ-Al_2_O_3_(100) at 873.15 K, for C_1_ adsorption. Color code as in previous figures.

Finally, the energy profile of Pt_5_/g-C_3_N_4_ under the same conditions is displayed in Figure 12 (C_1_) and Figure S6 (C_2_). The adsorption energy of propane is 146.6 kJ/mol, similar to that on Pt_5_/γ-Al_2_O_3_(100) (157.5 kJ/mol) and only slightly higher than on the naked Pt_5_ cluster (123.8 kJ/mol). The *E*_a_ of the first and second bond breaking are 26.0 and 12.2 kJ/mol, respectively, also falling between Pt_5_ (17.2 and 3.4 kJ/mol) and Pt_5_/γ-Al_2_O_3_(100) (37.1 and 24.3 kJ/mol). In addition, the desorption of H_2_ and propene for Pt_5_/g-C_3_N_4_ also falls between Pt_5_ and Pt_5_/γ-Al_2_O_3_(100). On the one hand, H_2_ desorption of Pt_5_/g-C_3_N_4_ is endergonic, but less energy (23.9 kJ/mol) is required, compared to that of naked Pt_5_ (50.7 kJ/mol). On the other hand, propene desorption of Pt_5_/g-C_3_N_4_ is exergonic, but less energy (47.8 kJ/mol) is released than on Pt_5_/γ-Al_2_O_3_(100) (92.9 kJ/mol). Catalyst activity and selectivity tend to go in opposite directions. Therefore, it is important that both are balanced. Thus, g-C_3_N_4_ may be a better support, as it improves the selectivity while balancing the activity.

**Figure 12:**
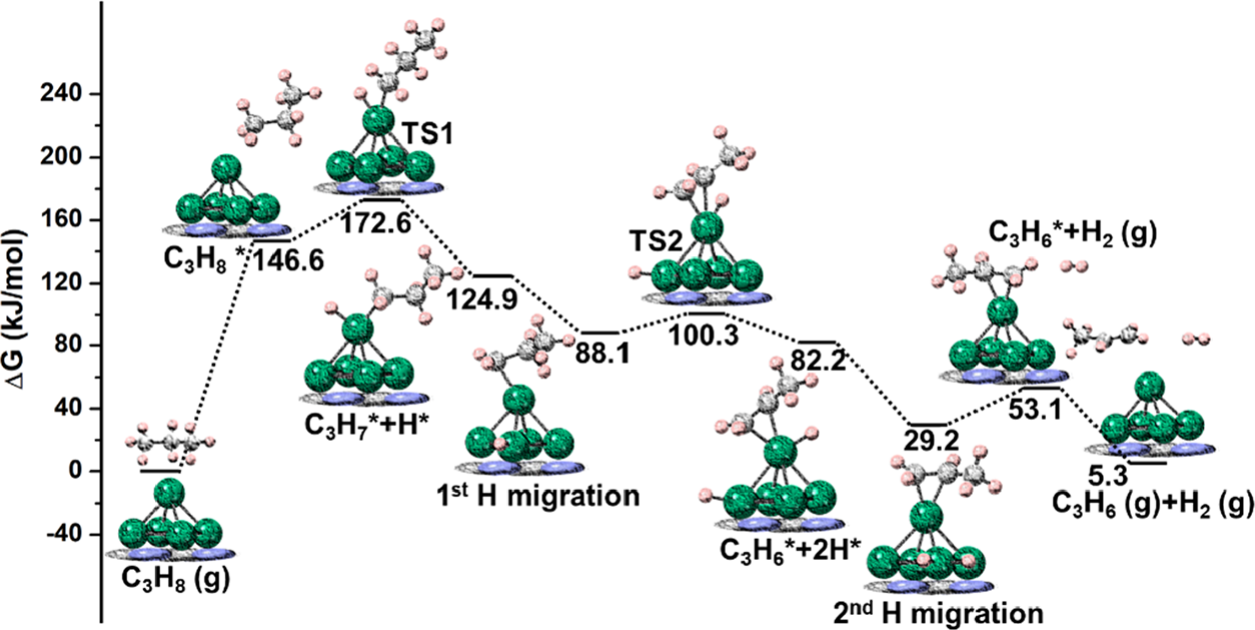
PDH free energy profile on Pt_5_/g-C_3_N_4_ at 873.15 K for C_1_ adsorption. Color code as in previous figures.

### Microkinetic Analysis of PDH on Naked and Supported Pt_5_

All of the previously considered reactions, as well as the elementary steps for C–C bond breaking of propane, C=C bond breaking of propene, and deep dehydrogenation (Table S3), were included in the microkinetic analysis. All catalysts reached equilibrium conversion at 873.15 K, ca. 33%, which is consistent with our previous results on Pt_1_ and Pt_4_/g-C_3_N_4_.^29^ In addition, the isolated Pt_5_ cluster has the highest activity at initial times (Figure 13) and then is overtaken by Pt_5_/g-C_3_N_4_. This is because of the high coverage of C_3_H_6_* + 2H* intermediates (about 97%) and C_3_H_6_* (about 0.8%) on the active site of Pt_5_, which corresponds to the cost of desorption (Figure 10). The coverage of C_3_H_6_* + 2H* on Pt_5_/g-C_3_N_4_ is about 22%, and no other intermediates are detected, which is consistent with the easier H_2_ desorption (Figure 12). The lower activity of Pt_5_/γ-Al_2_O_3_(100) is caused by the higher activation energy of TS1 and TS2 (Figure 11). Combined with the Gibbs free energy profiles, microkinetic analysis further confirms the best performance of Pt_5_/g-C_3_N_4_ among the three catalysts.

**Figure 13:**
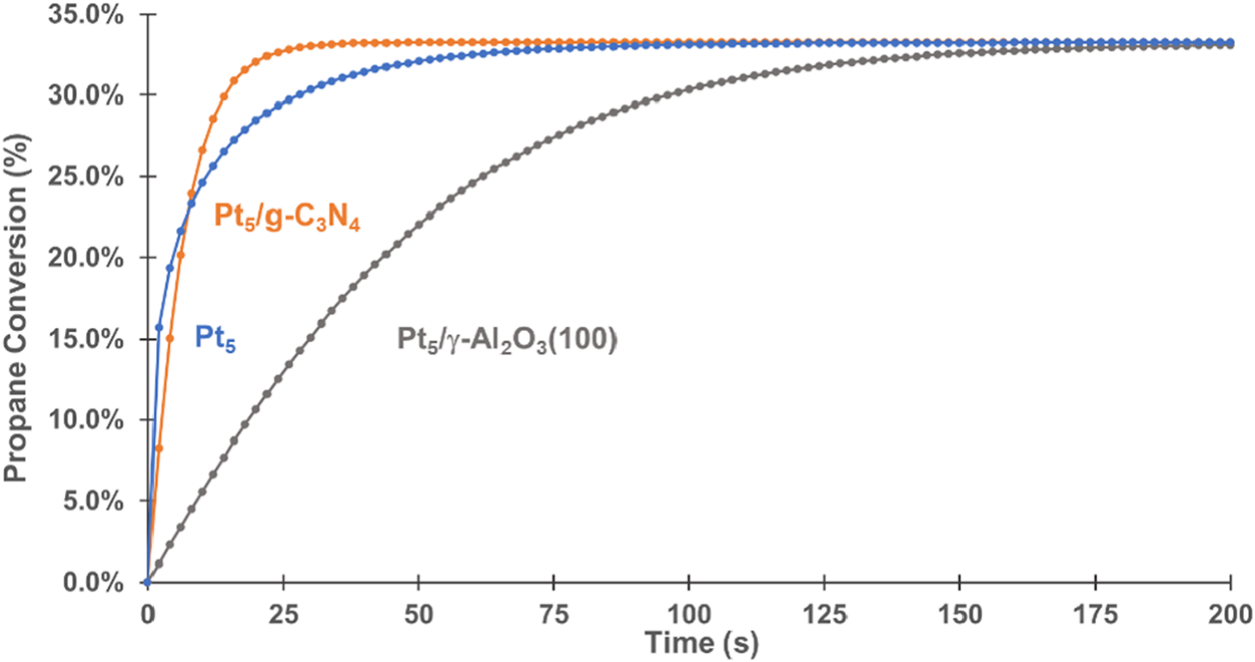
Predicted propane conversion on Pt_5_, Pt_5_/g-C_3_N_4_, and Pt_5_/γ-Al_2_O_3_(100) at 873.15 K, 1 atm.

We have also considered various temperatures around 873.15 K to estimate an apparent activation energy at initial times for the reaction following the Arrhenius equation. The results satisfied the linearity of the Arrhenius plot. The apparent activation energies were found to be 27, 40, and 49 kJ/mol for Pt_5_, Pt_5_/g-C_3_N_4_, and Pt_5_/γ-Al_2_O_3_(100), respectively. This is in agreement with the apparent activation energy of Pt_4_/g-C_3_N_4_ (57 kJ/mol), previously studied in our group.^29^ These estimations are also similar to the values obtained by Sui and Chen et al. for PDH on Pt/Al_2_O_3_ nanocatalysts, obtaining a value of 41 ± 5 kJ/mol for subnanometric clusters.^21^

In addition, the performance of Pt_5_/g-C_3_N_4_ was also compared with Pt_4_/g-C_3_N_4_.^29^ We found that Pt_5_/g-C_3_N_4_ was slightly more active than Pt_4_/g-C_3_N_4_ (Figure 14), according to the Gibbs free energy profiles (Figure S7).

**Figure 14:**
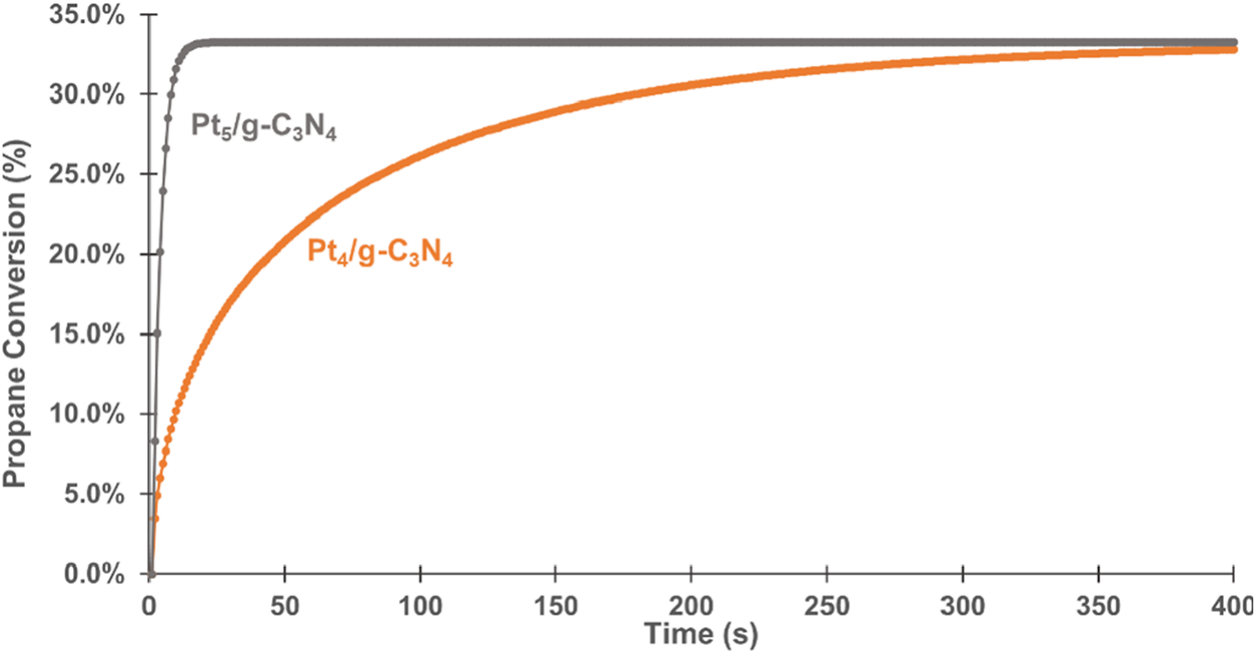
Predicted propane conversion on Pt_4_/g-C_3_N_4_ and Pt_5_/g-C_3_N_4_, at 873.15 K, 1 atm.

The profiles indicate that the free energy barriers for both Pt_4_/g-C_3_N_4_ and Pt_5_/g-C_3_N_4_ are similar, suggesting comparable activity for the C–H cleavage of propane. However, the desorption of H_2_ on Pt_4_ is more costly, due to the formation of a relatively deep energy well in C_3_H_6_* + H* + H* (2nd H migration step). In contrast, on Pt_5_, the desorption process is favored, leading to a better PDH performance.

## Conclusions

The performance of small Pt clusters, ranging from Pt_2_ to Pt_6_, as catalysts for PDH was investigated by means of theoretical calculations and compared with that supported on γ-Al_2_O_3_(100) and g-C_3_N_4_. The study reveals that both cohesion and interaction energies become stronger as the Pt cluster size increases, with the most stable surface–cluster interaction corresponding to adsorbed Pt_5_. The adsorption energies of propane on Pt*_n_*, Pt*_n_*/γ-Al_2_O_3_(100), and Pt*_n_*/g-C_3_N_4_ are comparable, resulting in similar energy barriers for the first and the second C–H bond breaking of propane (all barriers are lower than 47 kJ/mol). For the supported clusters, the adsorption energy of propene and hydrogen weakens when increasing the size of the Pt clusters until Pt_5_. At 873.15 K for Pt_5_, both desorption of hydrogen and propene are endergonic; while for Pt_5_/γ-Al_2_O_3_(100), both desorption processes are exergonic; and for Pt_5_/g-C_3_N_4_, the desorption of hydrogen is endergonic while the desorption of propene is exergonic.

Among the analyzed systems, both naked and supported Pt_5_ particles show the best performance in PDH in terms of stability, activity, and desorption. Moreover, the comparison of naked and supported Pt_5_ clusters reveals that the support is essential to tune the stability and desorption capacity of the catalysts. On Pt_5_/γ-Al_2_O_3_(100), desorption is easier (for both H_2_ and propene) than on Pt_5_/g-C_3_N_4_; however, the activation of propane follows the opposite trend, leading to the better PDH performance of Pt_5_/g-C_3_N_4_. Moreover, the performance of Pt_5_/g-C_3_N_4_ is also better than that of the previously studied Pt_4_/g-C_3_N_4_. The microkinetic analysis shows that deep dehydrogenation and C–C bond cleavage are rare events within the studied clusters, indicating a high selectivity for all of them.

## Supplementary Material



## Data Availability

All of the structures can be retrieved from the ioChem-BD database.^51^ (DOI: 10.19061/iochem-bd-2-83).

## Notes

The thermodynamic calculations, additional tables, and figures for the relevant structures and the C_2_ pathways. (PDF)
