# Supplementary material for: Propane Dehydrogenation Catalyzed by Pt Clusters (Pt2–Pt6) in Gas Phase and Supported on g‑C3N4 and γ‑Al2O3: A Theoretical Study
Source: ACS Omega. 2025 Sep 25;10(39):46105–14. doi: 10.1021/acsomega.5c07626 (PMC12509116; doi:10.1021/acsomega.5c07626)
Supplement: Supplementary file 1 [file ao5c07626_si_001.pdf]

Supporting Information

Propane Dehydrogenation Catalyzed by Pt Clusters (Pt_2-Pt_6) in Gas Phase and Supported on g-C_3N_4 and γ-Al_2O_3: A Theoretical Study

*Jie Pan, Gerard Bru, Jorge J. Carbó, Cyril Godard, Josep M. Ricart**

Departament de Química Física i Inorgànica, Universitat Rovira i Virgili, Tarragona, Spain.

Gibbs free energy from DFT (Page S2)

Microkinetic modeling (Page S2)

Tables

Table S1 Bader charges of Pt_n (n = 2-6) on γ-Al_2O_3(100) and g-C_3N_4. (Page S3)

Table S2 C_3H_6* + 2H* on Pt_5, C_1 adsorption. (Page S4)

Table S3 Reaction energies (Δ*E*) and activation energies (E_a) for all elementary steps in the reaction network of microkinetic analysis (Page S5)

Figures

Figure S1. E_{coh} (per atom) of NPs in Figure 1. (Page S5)

Figure S2. E_a (electronic energy) for the first (a) and second (b) C-H bond cleavage of propane (C_2 adsorption) in PDH. (Page S6)

Figure S3. E_a for the first C-H bond cleavage of propane (C_2 adsorption) (Page S6)

Figure S4. The energy profile of Pt_5 on PDH at 873.15 K, 1atm, C_2 adsorption. (Page S6)

Figure S5. The energy profile of Pt_5/γ-Al_2O_3 (100) on PDH at 873.15 K, 1atm, C_2 adsorption. (Page S7)

Figure S6. The energy profile of Pt_5/g-C_3N_4 on PDH at 873.15 K, 1atm, C_2 adsorption. (Page S7)

Figure S7. PDH free energy profiles on Pt_4/g-C_3N_4 and Pt_5/g-C_3N_4, at 873.15 K for C_1 adsorption. (Page S7)

References (Page S8)

Gibbs free energy from DFT

To consider temperature, we computed free energies (ΔG) following Eq. 1:

$$\Delta G = \Delta E_{ele} + \Delta E_{ZPE} + \Delta Cv_{harm} - T \quad (1)$$

where E_{ele} is the electronic energy, E_{ZPE} the zero-point energy, Cv_{harm} the heat capacity, and S the entropy.[1] E_{ZPE} (Eq. 2), Cv_{harm} (Eq. 3), and S (Eq. 4) are calculated using the vibrational frequencies (v_i).[1] which were obtained by constructing and diagonalizing the solution of the mass-weighted Hessian matrix under approximation of the harmonic oscillator. The frequencies were also used to characterize the minima and transition states. Then, the calculations of each item are as follows:

$$E_{ZPE} = \frac{1}{2}\sum hv_i \quad (2)l$$

$$Cv_{harm} = \sum_i^{harm} \frac{hv_i}{(e^{hv_i/k_BT}-1)} \quad (3)$$

$$S = k_B \sum_i^{harm} \left[\frac{hv_i}{k_BT(e^{hv_i/kT}-1)} - \ln(1 - e^{-hv_i/kT})\right] \quad (4)$$

It is worth noting that the commonly used harmonic approximation is supposed to be invalid when dealing with large-amplitude nuclear motions, resulting in unrealistic (too low) vibrational frequencies. Hence, the entropy may be over-evaluated because of the larger errors. To avoid this problem, we havelll replaced the vibrational entropy for all modes with frequencies <100 cm^{-1} by 100 cm^{-1}.[2]

Microkinetic modeling

The rate (r) of the elementary steps in the propane dehydrogenation (PDH) mechanism was determined by multiplying the coverage of reactants by the corresponding rate coefficient (k), equation 5.

$$r = k \cdot \theta_R \quad (5)$$

The rate coefficients were computed following the conventional transition-state theory of Eyring and Evans and Polanyi, as shown in equation 6.[3,4]

$$k = \frac{k_B \cdot T}{h} \cdot \frac{Z_{TS}}{Z_{IS}} \cdot e^{-E_a/k_B \cdot T} \quad (6)$$

where Z_{IS} and Z_{TS} are the partition functions in the initial and transition states, respectively. In this equation, E_a is the activation energy, T the temperature, k_B and h are the Boltzmann and Planck constants, respectively. As described by Laidler, ZPE is included in E_a.[5] Alternatively, the electronic energy can be used if the vibrational partition function is calculated with equation 7 that is referenced by McQuarrie. [6]. Thus, the vibrational partition function was calculated using equation 7, which includes explicitly the ZPE.

$$Z_{vib} = \prod_i \frac{e^{-hv_i/2k_BT}}{1-e^{-hv_i/k_BT}} \quad (7)$$

The rate of the adsorption steps was calculated using the classical Hertz-Knudsen equation:

$$r_{ads,i} = P_i(2\pi m_i k_B T)^{-1/2} A_{cat}\theta_i \quad (8)$$

where p_i is the partial pressure of the gas-phase species i, m_i is the molar mass, and A_{cat} is the area per active site.

This information was utilized to construct a microkinetic model, which involves solving a system of differential equations representing the reaction network composed of elementary steps, as shown in Table S3. The kinetic equations for each species form a reaction network that can be described by a system of Ordinary Differential equations (ODEs). These ODEs were solved numerically, given initial conditions, using computational methods. We employed the open-source Julia Programming Language to solve the reaction network, utilizing various packages, such as the DifferentialEquations.jl, which provides Rosenbrock methods to solving stiff problems.[7] This type of simulation offers the temporal evolution of species as a function of initial conditions, including the pressure of reactants and the temperature, similar to methodologies employed in previous studies.[8]

Tables

Table S1 Bader charges of Pt_n (n = 2-6) on γ-Al_2O_3(100) and g-C_3N_4. Al, O, C, N and Pt atoms are in blue, red, grey and lavender, respectively.

System	Side View	Pt_1	Pt_2	Pt_3	Pt_3	Pt_3	Pt_6
Pt_2/γ-Al_2O_3(100)	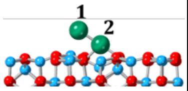	-0.7	0.3	—	—	—	—
Pt_3/γ-Al_2O_3(100)	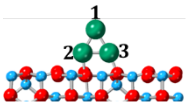	-0.2	-0.1	0.00	—	—	—
Pt_4/γ-Al_2O_3(100)	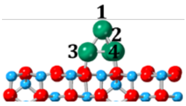	-0.1	-0.1	-0.2	0.2	—	—
Pt_5/γ-Al_2O_3(100)	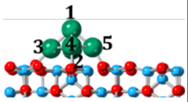	-0.1	0.0	-0.2	-0.2	-0.1	—
Pt_6/γ-Al_2O_3(100)	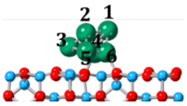	-0.1	0.0	-0.3	0.0	0.0	0.2
Pt_2/g-C_3N_4	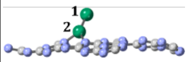	-0.4	0.4	—	—	—	—
Pt_3/g-C_3N_4	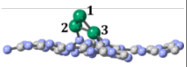	-0.3	0.1	0.4	—	—	—
Pt_4/g-C_3N_4	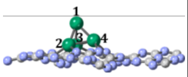	-0.2	0.3	0.3	0.2	—	—
Pt_5/g-C_3N_4	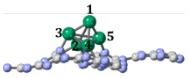	-0.2	0.1	0.4	0.1	0.2	—
Pt_6/g-C_3N_4	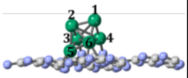	-0.1	-0.1	0.3	0.1	0.2	0.2

Table S2 C_3H_6* + 2H* on Pt_5, C_1 adsorption. C, H and Pt atoms are in grey, pink and green, respectively.

System	Adsorption mode	Structure	E_{ads} (kJ/mol)	ΔE (kJ/mol)
C_3H_6* + 2H* (C_1)	π adsorption	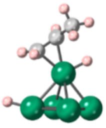	-162.7	0.0
C_3H_6* + 2H* (2nd H migration)	π adsorption	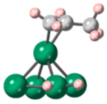	-193.7	-31.0
		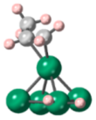	-194.9	-32.2
		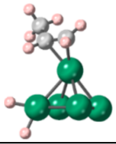	-195.3	-32.6
		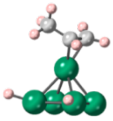	-202.1	-39.4
		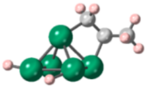	-202.4	-39.7
	σ adsorption	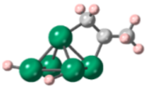	-151.1	11.6
		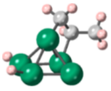	-159.1	3.6
		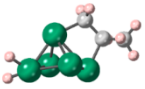	-142.7	20.0

Table S3 Reaction energies (ΔE) and activation energies (E_a) for all elementary steps in the reaction network of microkinetic analysis, all values are in eV.

Elementary step	Pt_5		Pt_5/g-C_3N_4		Pt_5/γ-Al_2O_3	
	ΔE	E_a	ΔE	E_a	ΔE	E_a
$CH_3CH_2CH_3(g) \rightleftharpoons CH_3{*}CH_2CH_3$	-0.48	—	-0.21	—	-0.28	—
$CH_3CH_2CH_3(g) \rightleftharpoons CH_3CH_2{*}CH_3$	-0.53	—	-0.25	—	-0.21	—
$CH_3{*}CH_2CH_3 \rightleftharpoons CH_2{*}CH_2CH_3 + H{*}$	-0.92	0.12	-0.36	0.54	0.29	0.73
$CH_3CH_2{*}CH_3 \rightleftharpoons CH_3CH{*}CH_3 + H{*}$	-0.87	0.20	-0.11	0.73	0.16	0.69
$CH_3{*}CH_2CH_3 \rightleftharpoons CH_3{*}+ {*}CH_2CH_3$	-0.40	0.86	0.15	1.27	0.41	1.39
$CH_3CH_2{*}CH_3 \rightleftharpoons CH_3{*} + CH_2{*}CH_3$	-0.34	0.91	0.18	1.30	0.34	1.32
$CH_2{*}CH_2CH_3 + H{*} \rightleftharpoons CH_2{*}CHCH_3 + 2H{*}$	-0.62	0.02	-0.76	0.21	-0.44	0.25
$CH_3CH{*}CH_3 + H{*} \rightleftharpoons CH_2CH{*}CH_3 + 2H{*}$	-0.60	0.01	-0.88	0.15	-0.34	0.22
$CH_2{*}CHCH_3 + 2H{*} \rightleftharpoons CH_2{*}CHCH_3 + H_2(g)$	1.53	—	1.79	—	1.13	—
$CH_2CH{*}CH_3 + 2H{*} \rightleftharpoons CH_2CH{*}CH_3 + H_2(g)$	1.51	—	1.71	—	1.09	—
$CH_2{*}CHCH_3 \rightleftharpoons CH_2CHCH_3(g)$	2.11	—	1.16	—	0.92	—
$CH_2{*}CHCH_3 \rightleftharpoons CH{*}CHCH_3 + H{*}$	0.91	1.34	0.78	1.23	0.40	1.10
$CH_2CH{*}CH_3 \rightleftharpoons CH_2C{*}CH_3 + H{*}$	0.89	1.33	0.38	0.80	0.38	0.94
$CH{*}CHCH_3 + H{*} \rightleftharpoons C{*}CHCH_3 + 2H{*}$	-0.05	0.47	-0.02	0.53	0.89	1.17
$CH_2C{*}CH_3 + H{*} \rightleftharpoons CHC{*}CH_3 + 2H{*}$	-0.52	0.28	-0.04	0.93	0.62	1.13
$CH_2{*}CHCH_3 \rightleftharpoons CH_2{*} + {*}CHCH_3$	1.54	2.26	2.43	2.68	2.15	2.98
$CH_2CH{*}CH_3 \rightleftharpoons CH_2CH{*}\ CH_3{*}$	0.21	1.40	0.07	1.27	0.23	1.61
$H_2(g) \rightleftharpoons H_2{*}$	1.45	—	0.62	—	0.26	—

Figures

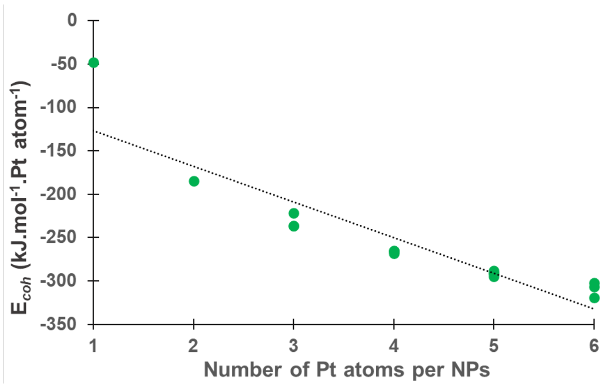

Figure S1. E_{coh} (per atom) of NPs in Figure 1.

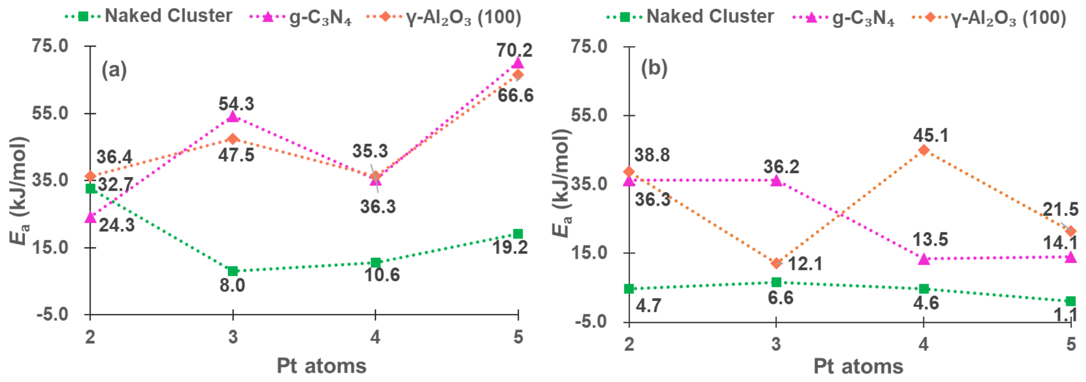

Figure S2. E_a (electronic energy) for the first (a) and second (b) C-H bond cleavage of propane (C_2 adsorption) in PDH.

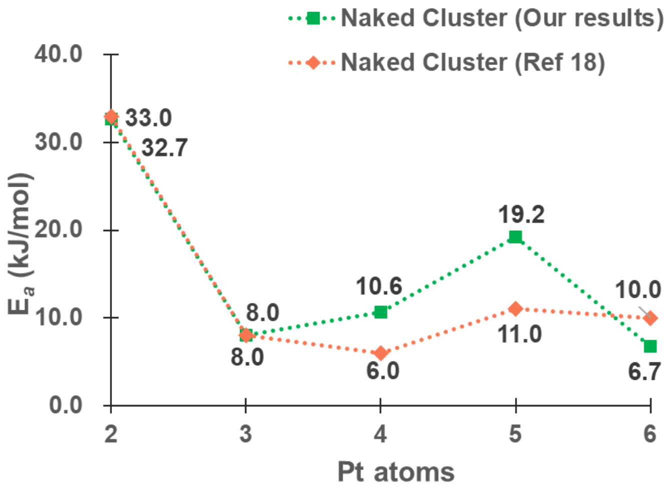

Figure S3. E_a for the first C-H bond cleavage of propane (C_2 adsorption), compared with Ref. 18.

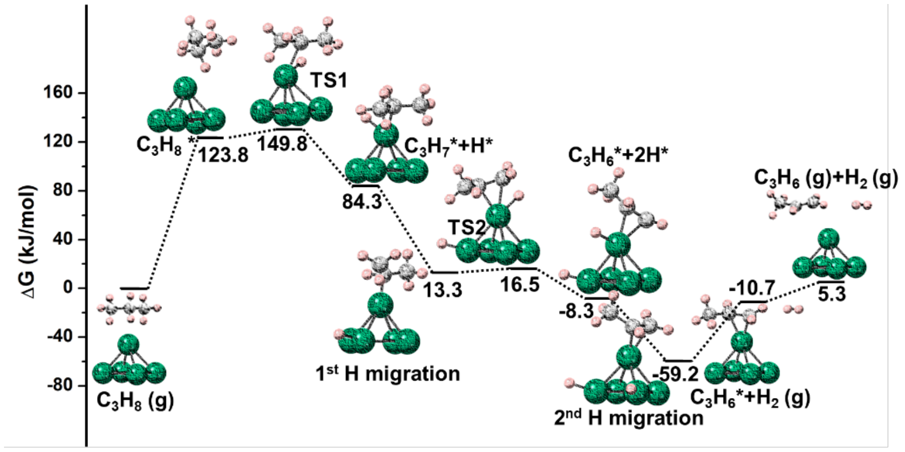

Figure S4. PDH free energy profile on Pt_5 at 873.15 K, for C_2 adsorption. Colour coding as in previous tables.

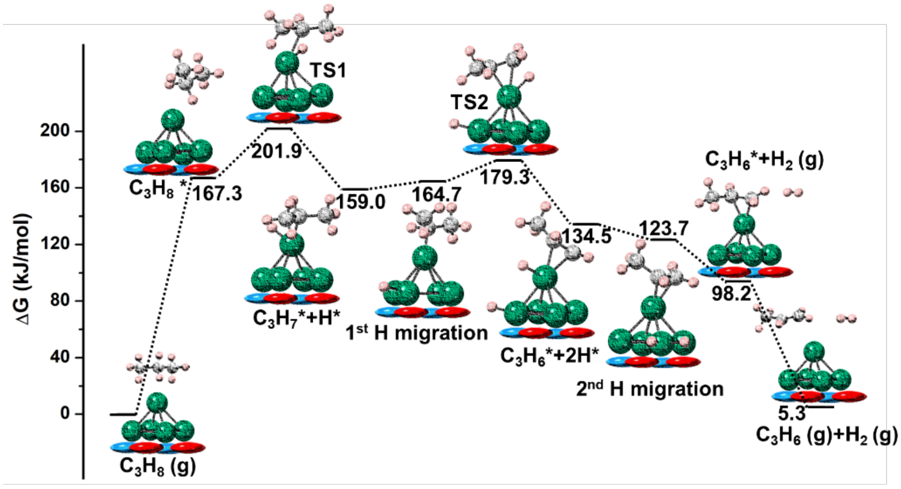

Figure S5. PDH free energy profile on $Pt_5/\gamma\text{-}Al_2O_3(100)$ at 873.15 K, for C_2 adsorption. Colour coding as in previous tables.

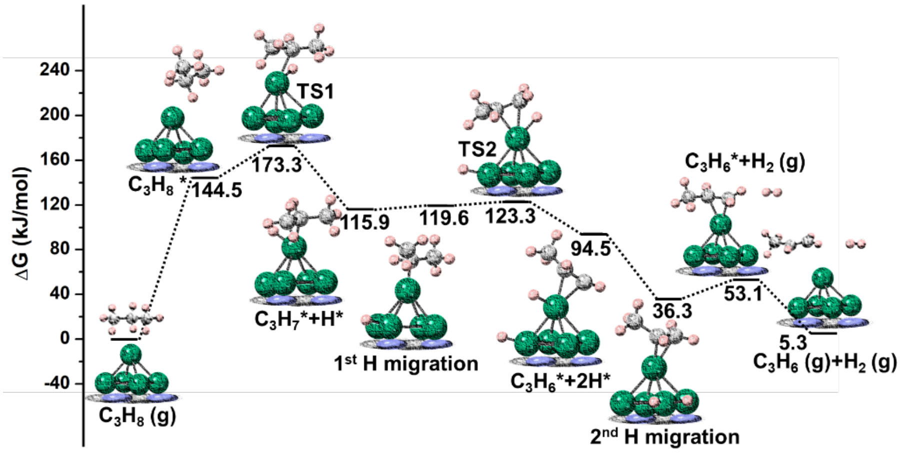

Figure S6. PDH free energy profile on $Pt_5/g\text{-}C_3N_4$ at 873.15 K, for C_2 adsorption. Colour coding as in previous tables.

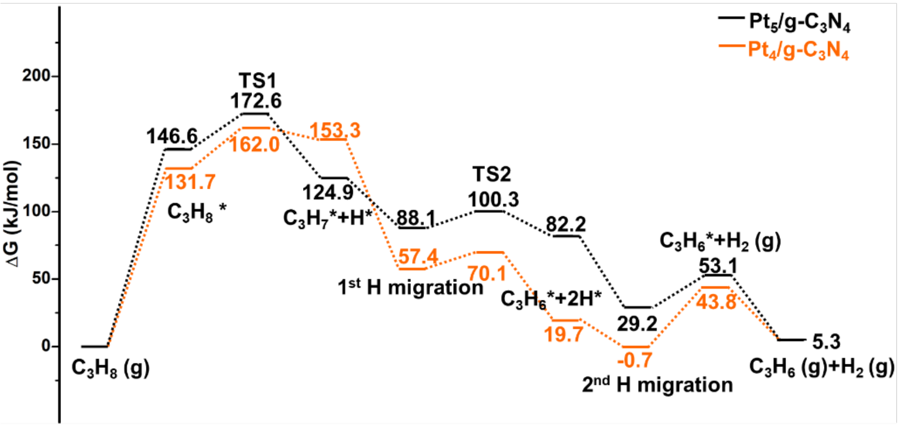

Figure S7. PDH free energy profiles on $Pt_4/g\text{-}C_3N_4$ and $Pt_5/g\text{-}C_3N_4$, at 873.15 K for C_1 adsorption.

References

(1) Cramer, C. J. Essentials of Computational Chemistry, Second Edition. Wiley, 2004

(2) Lozano-Reis, P.; Prats, H.; Gamallo, P.; Illas, F.; Sayós, R. Multiscale Study of the Mechanism of Catalytic CO_2 Hydrogenation: Role of the Ni(111) Facets. *ACS Catal.* **2020**, *10*, 8077-8089, DOI: 10.1021/acscatal.0c01599

(3) Eyring, H. The Activated Complex in Chemical Reactions. *J. Chem. Phys.* **1935**, *3*, 107-117, DOI: 10.1063/1.1749604

(4) Evans, M. G.; Polanyi, M. Some Applications of the Transition State Method to the Calculation of Reaction Velocities, Especially in Solution. *Trans. Faraday Soc.* **1935**, *31*, 875-895, DOI: 10.1039/TF9353100875

(5) Laidler, K. J. *Chemical Kinetics* Harper Collins: New York, 1987

(6) McQuarrie, D. A.; Simon, J. D. *Molecular Thermodynamics*, University Science Books, Sausalito, 1999

(7) Bezanson, J.; Edelman, A.; Karpinski, S.; Shah, V. B. Julia: A Fresh Approach to Numerical Computing. *SIAM Rev.* **2017**, *59*, 65-98, DOI: 10.1137/141000671

(8) Roldán, A.; Novell-Leruth, G.; Ricart, J. M.; Illas F. Pt(100) Theoretical Simulation of Temperature Programmed Desorption of Molecular Oxygen on Isolated Au Nanoparticles from density Functional Calculations and Microkinetics Models. *J. Phys. Chem. C* **2010**, *114*, 5101-5106, DOI: 10.1021/jp911283j
